# Use of a Novel, Digital Note‐Taking Application to Enhance the Learning of Lower‐Limb Anatomy Amongst Podiatry Students: A Qualitative Evaluation

**DOI:** 10.1002/jfa2.70190

**Published:** 2026-07-25

**Authors:** Neville Parker, Peter Roberts

**Affiliations:** ^1^ Division of Podiatry and Clinical Sciences The University of Huddersfield Huddersfield UK

**Keywords:** deep learning, digital, drawing, engagement, lower limb anatomy, podiatry

## Abstract

**Background:**

Learning anatomy is an essential part of the process of becoming a podiatrist. Anatomical knowledge underpins the assessment, diagnosis and management of lower limb pathology and competent professional practice. However, anatomy remains a challenging subject for students and educators. We have therefore developed an application to aid students in their continued learning and revision of anatomy in podiatric undergraduate training.

**Methods:**

This qualitative exploration sought the experiences and perceptions of six undergraduate podiatry students as they used the application alongside their traditional studies. Participants were provided with an iPad, a stylus and the necessary application and given 6 months to use the app. We convened a focus group to collect data on their experiences and opinions for how the app could be improved. Data were analysed using a reflexive, thematic approach.

**Results:**

Results were organised into two main themes: Engagement, and Improvements and Implementation and 5 sub‐themes: “Learning control and personalisation: a change from the norm” “Applicability to clinical practice” “Beyond anatomy” “Desired improvements, training and timing” and “Not just for independent learning”. These themes illustrated participants' positive experience with the app from an educational perspective as well as their suggestions for improvement.

**Conclusions:**

Participants engaged well with the note‐taking application and found learning from it enhanced their understanding of musculoskeletal practice in particular. They were positive about the independent learning opportunity it provided and could see uses beyond anatomy for future development. This project has enabled us to understand students' approaches to learning better and therefore be in position to develop the application more effectively for future use.

## 1 Introduction

This project took place at the University of Huddersfield in the Division of Physiotherapy and podiatry. Our aim was to introduce and evaluate a novel, digital approach to note taking and revision of lower limb anatomy to enhance engagement in developing core knowledge, and for continuous revision. We wanted to contribute to the understanding of technology enhanced teaching and learning through our specific focus on clinically important anatomical knowledge.

It is noteworthy that learning anatomy is an area of pedagogy which attracts attention for digitisation and deeper understanding of the effects of traditional approaches [1, 2]. It is evident from years of experience of both authors that this is an area of podiatric education which students find to be challenging, in particular the first‐year anatomy module on the undergraduate programme. Results for this module are significantly lower than those of other foundation level modules, where high proportions of students achieved grades above 60%. It should be acknowledged that this module requires a complex level of knowledge acquisition and is often a departure from preparatory subject students have studied prior to commencing the course. Podiatry education builds incrementally from foundational knowledge of anatomy to enhanced knowledge, which underpins learning regarding diagnostics and musculoskeletal care, for example. It is therefore essential that we support students in their learning of anatomy. Initial building blocks of anatomical knowledge are often challenging for students to solidify, making this a topic of high importance within teaching and learning.

In podiatric practice, deep and confident knowledge and understanding of both morbid and functional anatomy are essential. Anatomical knowledge of this kind is the foundation of safe and effective assessment, diagnosis, and management and is core to the undergraduate podiatry programme and HCPC standards of proficiency of “understanding and applying key concepts of the knowledge base relevant to the podiatry profession” [3]. Additionally, the largest UK professional podiatry body, the Royal College of Podiatry, includes anatomy knowledge within its musculoskeletal framework [4].

This project introduced and evaluated a novel approach to enhance learning and ongoing revision of anatomy for students. We developed foot and ankle anatomical templates for use in a mobile OS note taking application, which would enable students to draw and make notes on anatomical structures in a flexible way to support an integrated approach to learning and documentation in anatomy education. The app allows users to zoom and annotate with precision, add labels, text notes, audio recordings or additional images and diagrams. The free drawing, shape adding, and many other features enable students to add personalised drawings of muscles, tendons, ligaments, arteries, veins, and nerves to contribute to their learning.

Our aim was to explore the use of the app by a small group of students to understand their experience of use and opinion on areas for improvement. We wanted to understand if students felt the app might enable better understanding and retention of anatomy knowledge.

### 1.1 Knowledge Acquisition of Anatomy

The process by which students learn anatomy remains a topic of significant interest and investigation within the medical and allied health professions [5, 6]. Despite the importance of understanding how students learn in this field, there exists a notable gap in our understanding of what students perceive as beneficial to their learning of anatomy and how they effectively acquire anatomical knowledge. The pedagogic knowledge base relevant to podiatry is known to be limited [7] and performance in anatomy is noted to be affected by how students learn more broadly [8].

Various studies have shed light on the learning approaches adopted by students and their impact on academic performance [5, 6, 8, 9, 10, 11, 12, 13]. Research has consistently shown that students employing a deep learning approach, which emphasises understanding and visualisation, tend to outperform those utilising a surface approach, characterised by rote memorisation [8, 14]. Within the school education system and higher education, strategic approaches, that is students working to achieve the pass mark and little more, are perceived as the only way to survive, and perhaps the best way to perform conscientiously under conditions of high workload and competing demands [15, 16]. Anatomy in undergraduate podiatry teaching underpins large aspects of the podiatry curriculum and forms the foundation of future clinical practice [17]. Consequently, efforts continue to be made to reform curricula to promote deep learning strategies whilst recognising the prevalence of strategic techniques employed by students. The novel use of a note taking app in this way is one such approach.

Excessive focus on factual content can inadvertently encourage surface learning approaches, highlighting the need for holistic consideration of teaching methods and learning environments. In effect, deep approaches are associated with higher scores and surface approaches with lower scores [8]. Pandey and Zimitat [12] explored the quality of work and students' own perceptions of the best approach to learning anatomy and found that visualisation and understanding were associated with deep approaches to learning anatomy. Tailoring learning outcomes during dissection, promoting teamwork, and implementing regular assessments such as daily mini‐quizzes have all been associated with increased engagement and improved exam performance [10, 13, 18]. However, the extent to which these strategies promote deep learning versus surface learning remains a subject of debate [19].

Students studying anatomy for medicine, podiatry and pharmacy have similar approaches to learning anatomy and perspectives of their learning environment and that education should be flexible and accommodate various learning approaches [16]. Multimodal learning opportunities, including interactive activities, collaborative work, and regular assessments, may effectively cater to the diverse needs and preferences of students in this field. However, further research is needed to better understand the intricate interplay between teaching methods, learning environments, and student learning outcomes in anatomy education. Styn et al. [6] recently reported that tablet‐based drawing is a viable alternative to paper‐based methods for learning anatomy, and further exploration and research is needed to determine the most effective methods for podiatry students.

### 1.2 Enhancing Teaching and Learning Practices

The Podiatry academic team at the University of Huddersfield is committed to advancing teaching and learning practices for our students. To support this objective, we have created templates suitable for mobile tablets that can be used with digital note‐taking apps. The templates are intended to enhance course review outside of conventional lectures and to initially support students' revision.

Drawing and sketching have emerged as effective strategies for enhancing anatomy education by supporting spatial understanding, visualisation, and knowledge retention. Borrelli et al. [9] reported a 22.5% improvement in anatomical test scores and increased self‐confidence in students who engaged in drawing‐based learning. These findings suggest that incorporating visual and kinaesthetic elements can deepen comprehension. Although strong drawing skills enhance this benefit [20], even learners with limited proficiency can gain from guided templates [9], making this approach accessible and potentially cost‐effective in clinical education settings.

Alongside traditional methods, digital note‐taking platforms offer flexible, multimodal learning opportunities. These tools allow users to combine handwriting, typed input, and imported content, while offering organisational features such as tagging, categorisation, and search functions [21, 22]. Structured digital templates, in particular, have been shown to enhance the completeness and quality of notes, positively impacting academic performance in medical and health science students [18, 23]. While the transition to these tools can present usability challenges, they provide powerful opportunities for integrating drawing and note‐taking to reinforce anatomy learning [6, 24]. Despite their potential, the application of these methods to podiatry‐specific anatomy education remains underexplored, warranting further investigation into their effectiveness and relevance for this learner population. Borrelli et al. [9] suggest that this method of teaching and learning is engaging and encourages proactivity and that it is cost‐effective and can be easily integrated into the curriculum.

The purpose of this study, therefore, is to report on a novel approach to involving technologically literate podiatry students learning anatomy by using available technology available to students today.

## 2 Materials and Methods

This study utilised a descriptive phenomenological approach [25] to understand and learn from the experiences of students using the new application, descriptive phenomenology enabled us to explore the meanings participants attributed to their experience. The COREQ (Consolidated Criteria for Reporting Qualitative Research) has been used as a checklist to guide our write up [26]. We intended to explore participant experiences of using our application from a functional perspective in terms of using the technology alongside their perceptions of this as part of their learning experience. Ethical approval was gained through the institution's Research Ethics Committee, and permission to approach students was provided by the head of department (Ethics application reference SREIC/2024/013). Students in their 2nd and 3rd year of undergraduate BSc (Hons) podiatry studies were invited by email to volunteer to participate. A purposive sampling approach enabled us to recruit participants who were well positioned in their studies to provide rich data through an extended offer of participation to specific learners. The ethical application and approval covered concerns around ensuring non‐maleficence, and assurance that participation would have no influence over the study results as part of the purposive recruitment strategy.

We had six sets of equipment available for the project, and this was the main determinant in our sample size considerations. We did not consciously attempt to stratify participant selection, but the process did allow us to recruit from different age, ethnic and gender groups. We also had a mix of volunteers across our two main streams of undergraduate provision, the BSc (Hons) Apprenticeship and the traditional BSc (Hons) degree.

### 2.1 Identifying a Mobile Note Taking App

Participants were not directly involved in selecting the final note‐taking app. To inform the selection process, which included informing the procurement of devices, students from differing year groups were broadly canvassed about their use of tablets in their studies. Mobile note taking apps were reviewed following discussions with students about their preferences and any that were currently in use. Note‐taking apps are categorised into free (often limited), paid with free trials (typically offering basic features), and fully paid options. A search of peer‐reviewed literature revealed a lack of research on preferred apps among higher education students. Online review sites were analysed to highlight the advantages and disadvantages of various applications. The technology was evaluated, identifying key areas where digital note taking can be beneficial in educational settings. An adapted summary table, based on previous work [27], is provided Table 1. Notability was selected as a preferred app that some students were already using, with strategies developed to enhance its utilisation and features.

**TABLE 1:** Comparison of leading iOS note‐taking apps (adapted from Pitura, [27]).

Application	Price/plan model	Compatibility
Apple notes	Free (built in)	iOS, macOS
Evernote	Free + paid tiers	iOS, macOS
GoodNotes	Subscription/one‐time purchase	iOS, macOS
Microsoft OneNote	Free	iOS, macOS
Notability	Free starter + paid subscription	iOS, macOS
Notion	Free + paid plans	iOS, macOS

### 2.2 Development of the Templates

**FIGURE 1:**
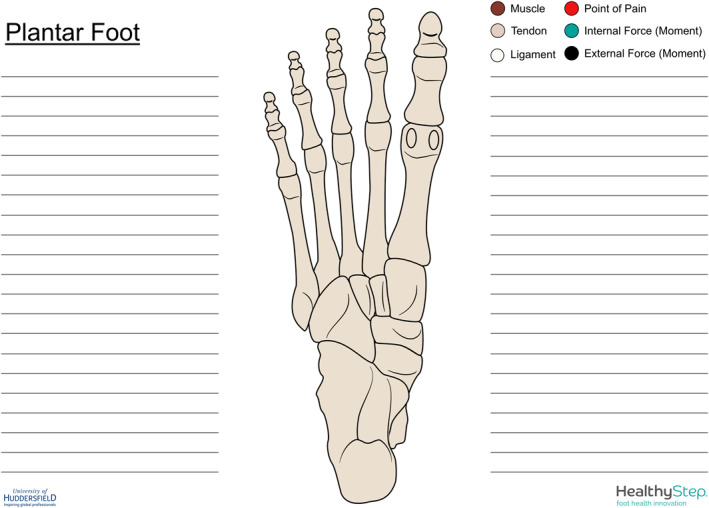
Example of template; Plantar foot depicted.

The undergraduate podiatry programme introduces lower limb structure and function at foundation level and this builds into subsequent years. At intermediate level, musculoskeletal pathology is taught and considered in regions such as, the anterior knee, or medial ankle. Therefore, the templates were developed to align with the programme theme of learning anatomy and MSK assessment by region. A limited number of templates were developed to not overwhelm students or affect their ability to commence using them. The following templates were made available to the students: Medial/Lateral ankle & foot, Dorsal/Plantar Foot, Anterior/Posterior Foot, Anterior/Posterior knee. An example of a template is shown in Figure 1.

Participants were provided with an iPad (iPad 10th GEN, A2696, Wi‐Fi, 64GB) and a stylus (Tucano Active Stylus Pencil, MA‐STY‐W). They received instruction on how to use the app and templates and were then given 12 weeks to use the equipment as a supplement to their ongoing studies.

### 2.3 Data Collection

At the conclusion of the trial period, participants were invited to attend a focus group interview held via Microsoft Teams. Participants were asked to discuss their feedback on the technical use of the application in terms of ease of use and areas for improvement. From this perspective, the discussion provided data to inform enhancement of the application. Other discussions were directed towards students' experiences of using the application as part of their learning experience. A semi‐structured approach enabled the participants to share perceptions and stories of how they had used the application to inform their learning. The semi‐structured questions used for the focus group are detailed in Figure S1: Appendix A. Discussion topics were led and prompted by both researchers to understand participant experiences and learn about their suggestions for improvement. To counteract potential negative power dynamic, we facilitated freedom of discussion, reiterating our ethical commitments to exploring participant experiences with no implications for their studies or future grades. The group nature of the discussion provided opportunity for corroboration and expansion as the group probed and quizzed each other on ways in which they had each used their time with the equipment alongside their traditional studies. The focus group interview was 45 minutes in duration, providing the group with sufficient time and opportunity to fully explore their experiences of using the application.

In addition to the focus group, the researchers planned a contingency data collection method of a written, qualitative survey. We intended this to mitigate against participants being unable to attend the focus group for unforeseen reasons or work commitments. A set of questions following the same approach and aims but organised for written responses was issued to participants in the event of them being unable to attend which are detailed in Figure S2 Appendix B.

Microsoft Teams produces a transcription of the meeting in MS Word format. This transcription was checked for accuracy by both researchers. Both researchers then immersed themselves in the data before commencing formal analysis using a thematic approach after Braun and Clarke [28]. Microsoft Teams recording provided video from the call and this was preserved for use during analysis for observation and to aid clarity of understanding meaning. Both authors followed the process of familiarisation, Coding, forming initial themes, developing and reviewing themes and finally refining and defining the ultimate themes. Analysis was undertaken reflexively and with dialogue between the researchers. This was important given that both NP and PR are white, male, middle aged podiatrists with experience in clinical practice and education. The authors questioned their conclusions and discussed with each other to ensure preconceptions and biases were considered during analysis and interpretation. The process was iterative and flexible to ensure trustworthiness at each stage. Data analysis was the same for both the focus group and the written survey responses.

## 3 Results

Three participants took part in the focus group, and the remaining participants provided written feedback because they were unwell or unavailable and could not attend. Participants' real names have been replaced with pseudonyms. Participant demographic details are shown in Table 2.

**TABLE 2:** Participant demographics.

Pseudonym	Gender	Age	Year of study	Route of study	Response method
Alex	Male	35	3	BSc (Hons) Apprenticeship	Focus group
Beth	Female	25	3	BSc (Hons) level 6 podiatry Apprenticeship	Focus group
Aminah	Female	20	2	BSc (Hons) podiatry	Focus group
Cho	Female	19	2	BSc (Hons) level 6 podiatry apprenticeship.	Survey Response
Caroline	Female	28	3	BSc (Hons) podiatry	Survey Response
Saira	Female	22	3	BSc (Hons) podiatry.	Survey Response

### 3.1 Themes

**FIGURE 2:**
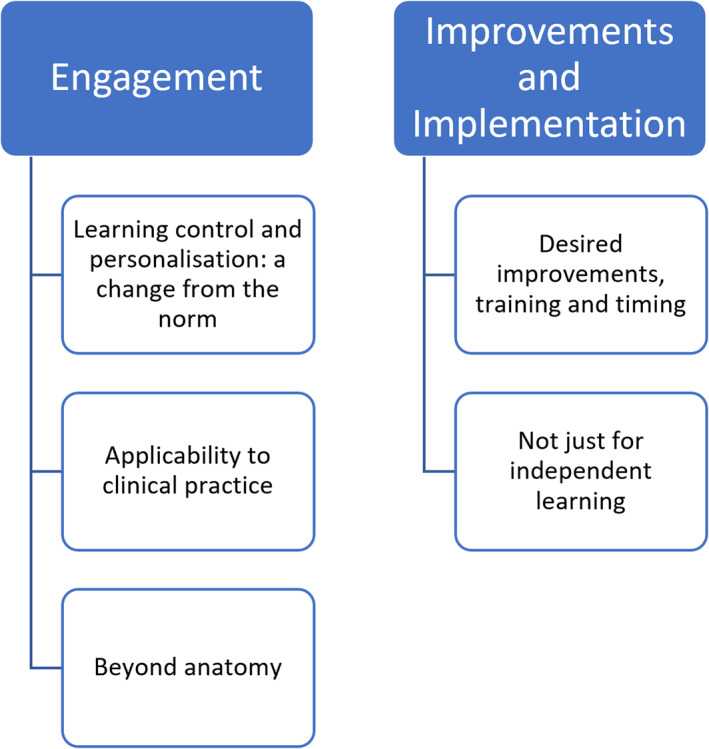
Themes extracted from the focus group.

Two main themes and five sub‐themes were identified following analysis. Multiple iterations of thematic structure were considered and reviewed until the researchers considered all viewpoints and data had been represented. The themes are presented in Figure 2.

### 3.2 Engagement

Engagement is a complex but important concept in Teaching and Learning. It is essential when developing skills, knowledge and professional identity [7, 29]. The latter element being of relevance with the centrality of anatomy knowledge to podiatry practice. Measurement and definition of engagement is highly varied and often reduced to counting student interactions with virtual learning platforms or monitoring attendance for example. We were interested in how the app was used by students and whether this was something they felt enhanced their ability to learn. The data provided insight into participants' engagement with learning material through the technology and app. This goes beyond the act of attending a lecture as evidence of engagement; it includes information about how participants manipulated images, words and sources to align with their individual learning styles. Based on this understanding of engagement we have identified sub themes of Learning Control and Personalisation: A change from the norm, and Applicability to Clinical Practice.

#### 3.2.1 Learning Control and Personalisation: A Change From the Norm

Aspects of learning styles and the control afforded by the tablet and drawing app constituted a significant theme of discussion. Participants enjoyed the flexibility to continue their learning away from the classroom in a way which complemented what had been delivered. In the focus group, this was expressed as the app enabling better ownership of the notes:

It was nice to have your own control about your notes. the layout, the format, what you wanted to focus on. That was good. Being able to put things that you’ve found, like the research and MSK knowledge, into your own words was brilliant.

(Cho)

The pen provided was great also, as the action of physically drawing the nerves enabled me to commit to memory more easily. I often make notes on paper for that reason, so it was incredibly convenient to have everything in one place.

(Aminah)

The advantage of this, as articulated by the group, was that combining pictures with their own interpreted notes meant students could understand and retain information more easily:

…because it's alright having paragraphs and paragraphs and articles and journals and research behind why something happened, but being able to pinpoint that on its own section for visualising what I’ve drawn and what I’ve written.

(Cho)

The drawing app was separate from the material delivered in class, and this was seen emphatically as an advantage to student learning. It was an opportunity for them to control their handling of the information in one place. Further to this, it also facilitated retention of knowledge through repetition of action to commit structures to memory. This strong shared feeling was evidence of the control students could exercise over their learning through the application:

I spent quite a lot of time just drawing and redrawing muscles and arteries and things, then labelling them up, rubbing them out, labelling them up again, and so on.

(Alex)

Views amongst the participants revealed that this was a refreshing change from what they had expected or already experienced in their own independent study. They were enthused and engaged by the versatility of the application when it came to their individual requirements.

That's something that I that's something that I really struggled with in the first year is finding my way of learning. So, I thought it was just writing and writing and writing over and over again, the same thing on multiple pieces of paper, when actually, you know, mind maps can be used in the app, drawing, colouring, highlighting that that all sort of like condenses into one when you're using it.

(Beth)

#### 3.2.2 Applicability to Clinical Practice

As stated in our background and literature, the importance of podiatry students achieving deep working knowledge of anatomy cannot be overstated. To become confident in the independent assessment, diagnosis and management of MSK conditions of the foot and ankle requires a firm foundation knowledge of anatomy. Participants made clear links between their use of the app and its application in clinical practice:

I do have an example, Plantar plate tears. I had a patient with one in work the other day, and I was never confident on plantar plates until I was able to draw on the diagram and then put next to it, plantar plate tears are caused by XY and Z.

(Beth)

Indeed, without the tablet in clinic, the participants described their experience of how the app had increased their confidence in anatomy knowledge to extend helping make MSK diagnoses:

Repeatedly drawing, redrawing, learning and making sure everything is in the right place in my head, it helps with patients, especially with MSK patients who can pinpoint the area that might be painful or causing a problem

(Saira)

For the students participating in this research, the iPad offered an additional means of narrowing the theory‐practice gap and enabling them to visualise injured structures and develop a better working knowledge of how that may link to making diagnoses and planning treatments.

#### 3.2.3 Not Just Anatomy

Our templates were designed with anatomy learning in mind, and final year (honours level) students highlighted that this would be of significant benefit to underpin the learning of musculoskeletal podiatry in the second year. Such is the requirement for deep anatomical knowledge for that subject. In addition, it was emphasised by all participants that the application would work beyond anatomy and be applicable to other learning, such as dermatological conditions:

Probably even pod theory one in first year would have been perfect because you can draw corns and calluses and what stresses cause it. You can put it all on the drawing.

(Caroline)

The potential they saw extended to high‐risk areas of practice and highlighted not only the anatomical level of drawing but also what they could add from a clinical management perspective. Wound care and the planning of offloading and dressing strategies was one such area considered as fitting with the style of learning the application afforded.

Maybe going into wound care, possibly. You know, this is where you've got your neuropathic ulcer. So can you move this and what [shape] would you use semi compressed felt, which dressings and moving them to the area that kind of thing? I just think you'd be able to use it in pretty much every module.

(Alex)

### 3.3 Improvements and Implementation

Discussions and written feedback provided valuable information for development and enhancement of the templates. The qualitative nature of our enquiry allowed participants to expand on their experiences and share opinions on how they believed the approach could be used across other areas of their learning.

#### 3.3.1 Desired Improvements: Training and Timing

One area of desired improvement arose from the students finding that they wanted more space to use the functionality. Their positive experience of combining pictures with notes led them to want to be even more creative and expressive with their note keeping. They reported more space would facilitate this:

Space to write on the outside, so possibly moving it to like an A6 layout if that makes sense. You've still got, you've still got your diagram in the middle, but just being able to extend how much space you have to write. – More white space around the templates

(Alex)

Conversely, other participants found the option to zoom and edit pictures with text gave them a sufficient canvas for their learning. They did, however, note that there was considerable subjectivity between what different users prefer, and so ultimately some optionality would be desirable.

Discussions revealed a desire for some more training on the use of the application so that students would be able to get using it more quickly and reap the benefits. It was also suggested that the flexibility of learning functionality would lend itself to peer coaching and collaboration. The feeling was that this would bring about more effective use of the application as well as sharing learning.

But there's things I saw on there that I didn't know how to do. Or maybe if there was someone or like if we all came together as a group and showed each other what we learn.

(Beth)

The need for guidance and demonstration of different ways of learning emerged as an additional discussion. Participants were positive about using the iPads and engaging with the application for anatomy, but extrapolated their need for training with the technology to other areas of learning. This would include such things as revision techniques, library resources, group work opportunities and effectively some guidance on how best to learn.

But younger people coming into university with all of this wealth of information that's there to be learned might not be coming up with that knowledge of how it works best for them. So, maybe a tutorial session showing what is available and what they can do and how they can learn I think would benefit everyone, not just in general.

(Alex)

#### 3.3.2 Not Just for Independent Learning

Participants felt the technology and applications could work in taught sessions as well as for independent study. Such was their engagement with the design that it was positive to note their confidence in expanding its availability and use.

The lecturer could display the template and, rather than just talking through with, you know, static images, what's there, the lecturer could draw on their own that would then display on the projection and on the students' tablets as well, and they could then access.

(Saira)

Participants in this sense were thinking about factors including accessibility of information in learning sessions, perhaps for students with learning disabilities. There was also a clear sense that the technology could further move away from the use of paper resources and thus provide a sustainable and environmentally friendly element.

The environmental and printing costs would also help if you could just grab an iPad and look up the worksheet rather than you guys have to print these off and hand them out.

(Cho)

## 4 Discussion

The focus group discussion has revealed how students were engaged with the novel use of the app for learning anatomy to the extent that it had both increased their factual knowledge and their application of that knowledge in practice. This represented evidence of a movement from superficial or propositional knowledge to deep, applied knowledge. This is a distinction previously recognised as highly important in developing effective graduate professionals [30].

The use of a flexible, digital, drawn approach appears to have made understanding anatomy and subsequently musculoskeletal podiatry less of a technical challenge and more of an applied process. The main theme of Engagement was so named because the participants embraced and used the novel approach in ways which suited their learning styles, and which they recognised as being beneficial to their understanding. Entering the podiatry profession requires more than just attaining the level commensurate with HCPC and Royal College of Podiatry standards. Graduates are transforming into professionals. Previous work from Tobbell and Roberts [7] modelled this process as having three elements: *Imagination, Alignment and Engagement*. In our study, we have noted that engaging with the app enabled participants to imagine how they would use their newly gained knowledge in practice. They had drawn a sense of confidence from this and seen it as a bridge into their practice.

Significantly, there was a positive emphasis on the option to draw and re‐draw in a way that was environmentally sustainable and focused on their requirements. Participants in this project had already achieved a pass in the foundation year anatomy module; therefore, their use of the app was not linked to a strategic approach of attempting to simply gain academic credit. Instead, these students were engaged in developing their deep learning and understanding and reflected on it in that sense. This echoes work by Barbian et al. [31] who found active and collaborative approaches to learning anatomy enhanced performance over more structured and passive methods. Indeed, our participants saw the opportunity to do the same beyond anatomy focussed topics. This contrast with surface learning approaches of doing what is needed to fulfil course requirements was a finding which we see as positive to fuel future development of this new approach. When compared to qualitative medical, literature [32] it is interesting to note that our participants found some security in their anatomical knowledge had developed since using the app. Although independent of direct structured education this was learning that directly and positively enhanced their understanding and experience.

The act of drawing and sketching, which is guided and linked with note keeping was perceived to be beneficial to learning. This is in accordance with literature outside of podiatry [9, 21, 22] and demonstrated the control that the app gave students over their learning. For students with different learning styles of specific learning disabilities, this control is important in reducing the differential attainment, which is sometimes evident. Although this study did not set out to test the impact of the app on summative academic attainment, we were encouraged by the participants' shared positive view that this was a good aid to study and revision. However, we are not naïve to ignore the possibility of a Hawthorne effect and would expand the study to explore cause and effect in terms of the superficial learning elements in the first instance. From a reflexive perspective, we needed to acknowledge our intention of introducing this as a positive development, and we ensured to ask for negative experiences and areas for improvement.

We learned a great deal in terms of future improvements. We were impressed by the imagination participants showed when considering implementation and wider roll out. Participants shared their experience of learning the new technology and extrapolated their needs to wider issues around study support and assistance in learning how to learn. This is an incidental finding, but one which will be considered and reviewed across the course and not just in rolling out our new development. Kruse et al. [33] shared strategies on confronting misconceptions amongst students regarding learning only being classroom based. We believe use of a note taking app in this way is one of many ways in which these misconceptions can continue to be broken down and true self‐directed, deep learning can be encouraged. It was a positive and unexpected finding of our exploration. Indeed, such was participant independence and engagement with the app that they had found their own ways around many of the initial perceived limitations. So much so that one of the recommendations for future roll out was to establish peer to peer support groups to share in best use of the devices and app.

### 4.1 Study Limitations

This qualitative study was conducted with the aim of exploring and understanding students' experiences of using this novel approach to supporting their learning and revision of anatomy. We also wanted to access their opinions on how our approach could be improved. For these reasons, the study is not intended to be generalisable but should enable those interested in the study of pedagogical practices and technology enhancement within Allied Health Professional education to see how the field is advancing. We are satisfied with our sampling and sample size. We were disappointed to not have more voices in the focus group to contribute to the generation of multiple viewpoints through dialogue. However, the written survey data aligned with our methodology, and we are confident that this is a novel contribution to the growing arena of technology enhanced teaching and learning research.

## 5 Conclusions

The intention of all pedagogic research within health subjects is that it will ultimately benefit the public, patients and the profession via increased confidence and ability of students during and after their undergraduate training. Whilst it is beyond the reach of this study to establish such a finding, we are encouraged that this example of a novel, digital approach to support student learning and revision has been experienced by students to be of potential benefit to their care of patients. It is evident from our data that participants recognised the potential for this application to facilitate deep learning as an adjunct to their traditional study. Enhancing student knowledge of anatomy throughout their degree studies should positively affect concurrent learning and development of professional identity and clinical skills such as diagnostics and musculoskeletal care. Further research on the learning journey of podiatry students and the progressive nature of anatomy knowledge acquisition is required. Additionally, suggestions from our participants that this approach could be used more widely and embedded into the course at an earlier stage require further exploration. This study has provided a baseline of experiential data and a positive basis for further development. We suggest there is opportunity to expand exploration of this digital learning approach to other Higher Education Institutions and professional groups both nationally and internationally.

## Author Contributions

**Neville Parker:** conceptualization, funding acquisition (equal), data curation (equal), formal analysis (equal), methodology (supporting), investigation (equal), writing – original draft (equal), writing – review and editing (equal). **Peter Roberts:** funding acquisition (equal), data curation (equal), formal analysis (equal), methodology (lead), investigation (equal), writing – original draft (equal), writing – review and editing (equal), supervision.

## Funding

This project was partly supported by funds from the Royal College of Podiatry charitable fund for the purchase of 6 iPads, styluses and cases for participants to use for the duration of the study. This was obtained through competitive grant capture.

## Ethics Statement

Ethical approval is detailed within the text and included participation and publication of quotes under pseudonym.

## Consent

Consent for publication was gained from all participants as part of the institutional ethical clearance process.

## Conflicts of Interest

The authors declare no conflicts of interest.

## Supporting information


**Figure S1:** Focus group semi–structured interview questions.

**Figure S2:** Questions asked of participants unable to attend the focus group in the form of a written response.

## Data Availability

The data that support the findings of this study are available from the corresponding author upon reasonable request.
