# Supplementary figures and images for: Use of a Novel, Digital Note‐Taking Application to Enhance the Learning of Lower‐Limb Anatomy Amongst Podiatry Students: A Qualitative Evaluation
Source: J Foot Ankle Res. 2026 Jul 25;19(3):e70190. doi: 10.1002/jfa2.70190 (PMC13401503; doi:10.1002/jfa2.70190)

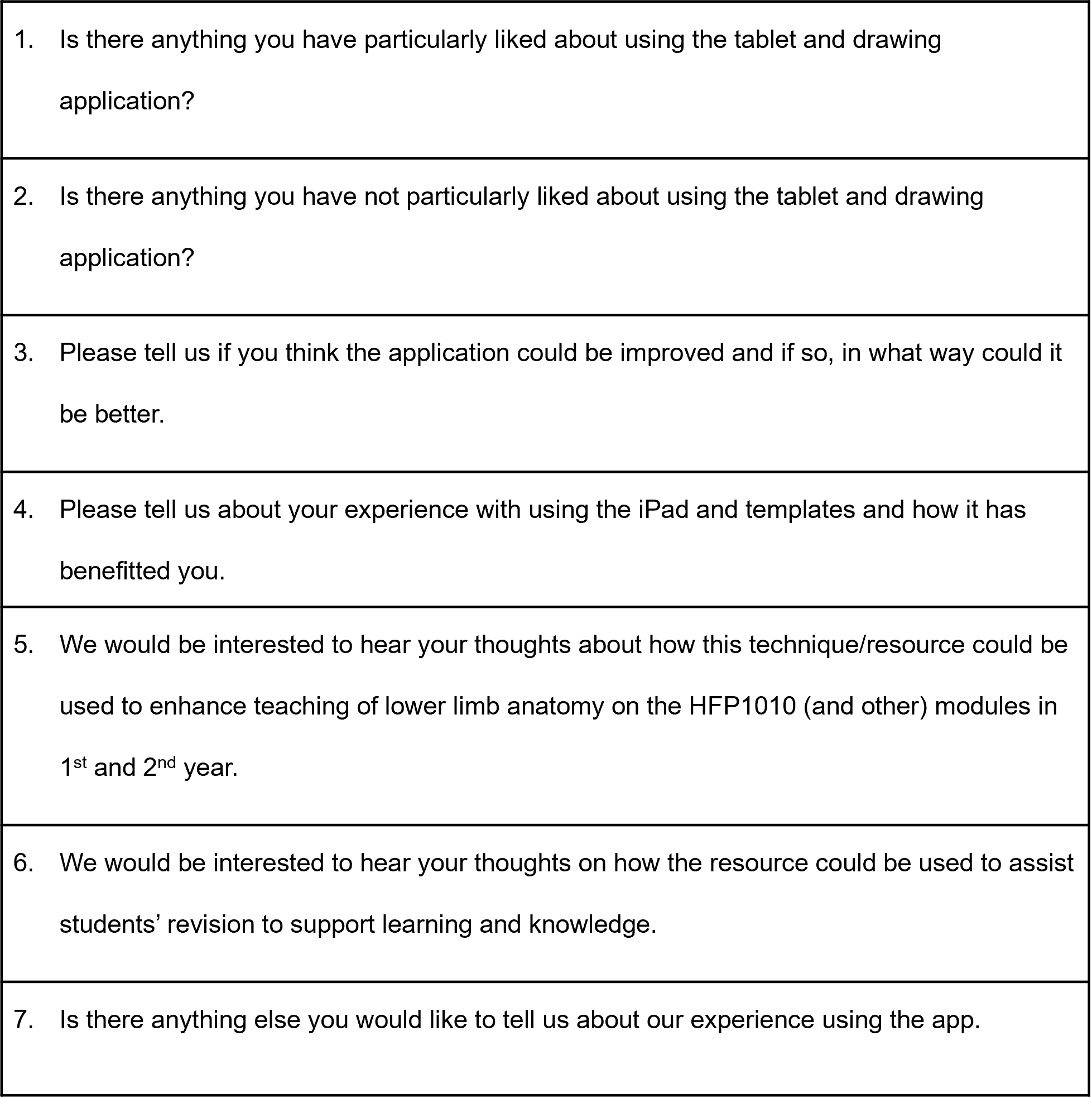

Supplement: Supplementary file 1 — Figure S1: Focus group semi–structured interview questions. [file JFA2-19-e70190-s002.png]

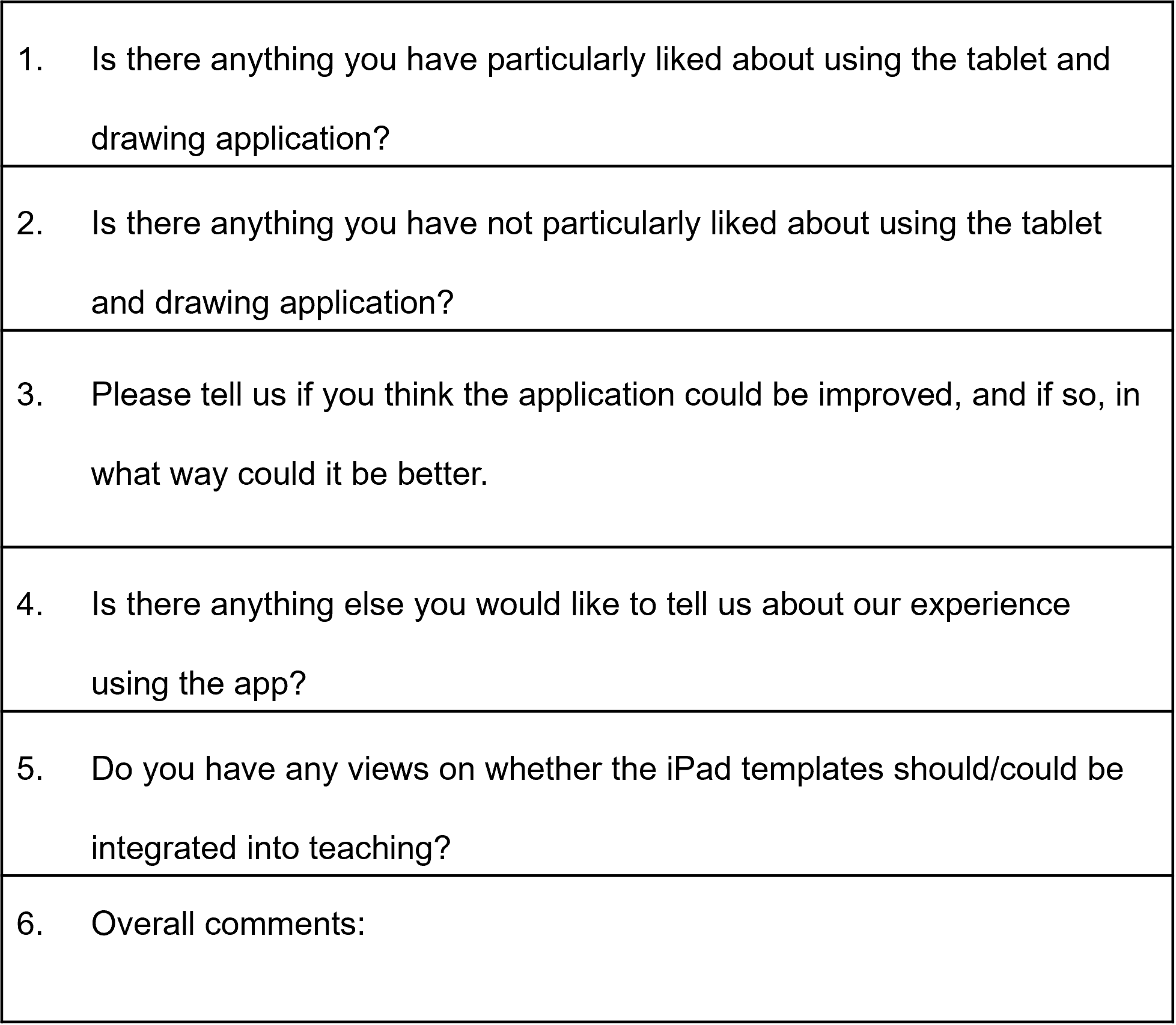

Supplement: Supplementary file 2 — Figure S2: Questions asked of participants unable to attend the focus group in the form of a written response. [file JFA2-19-e70190-s001.png]
